# Distinct Roles of TRAPPC8 and TRAPPC12 in Ciliogenesis via Their Interactions With OFD1

**DOI:** 10.3389/fcell.2020.00148

**Published:** 2020-03-17

**Authors:** Caiyun Zhang, Chunman Li, Gavin Ka Yu Siu, Xiaomin Luo, Sidney Yu

**Affiliations:** ^1^School of Biomedical Sciences, The Chinese University of Hong Kong, Sha Tin, China; ^2^Department of Anatomy, Histology and Developmental Biology, School of Basic Medical Sciences, Shenzhen University Health Science Centre, Shenzhen, China

**Keywords:** centriolar satellite, OFD1, primary cilium, TRAPPC8, TRAPPC12

## Abstract

The transport protein particle (TRAPP) complex was initially identified as a tethering factor for COPII vesicle. Subsequently, three forms (TRAPPI, II, and III) have been found and TRAPPIII has been reported to serve as a regulator in autophagy. This study investigates a new role of mammalian TRAPPIII in ciliogenesis. We found a ciliopathy protein, oral-facial-digital syndrome 1 (OFD1), interacting with the TRAPPIII-specific subunits TRAPPC8 and TRAPPC12. TRAPPC8 is necessary for the association of OFD1 with pericentriolar material 1 (PCM1). Its depletion reduces the extent of colocalized signals between OFD1 and PCM1, but does not compromise the structural integrity of centriolar satellites. The interaction between TRAPPC8 and OFD1 inhibits that between OFD1 and TRAPPC12, suggesting different roles of TRAPPIII-specific subunits in ciliogenesis and explaining the differences in cilium lengths in TRAPPC8-depleted and TRAPPC12-depleted hTERT-RPE1 cells. On the other hand, TRAPPC12 depletion causes increased ciliary length because TRAPPC12 is required for the disassembly of primary cilia. Overall, this study has revealed different roles of TRAPPC8 and TRAPPC12 in the assembly of centriolar satellites and demonstrated a possible tethering role of TRAPPIII during ciliogenesis.

## Introduction

Primary cilia are microtubule-based sensory organelles that project from the surface of most mammalian cells (Malicki and Johnson, 2017). They recognize signals from the extracellular environment and control multiple intracellular signaling pathways (Downs et al., 2014; Malicki and Johnson, 2017). Defects in ciliary structure and/or function cause developmental and physiological disorders collectively called ciliopathies, which include Bardel Biedl syndrome (BBS), orofaciodigital syndrome (OFD), Meckel Gruber syndrome (MKS), nephronophthisis (NPH), retinal degeneration (RPGR), and Joubert syndrome (JS) (Adams et al., 2007; Lopes et al., 2011; Hemachandar, 2014; Novas et al., 2015). These diseases are characterized by their genetic heterogeneity which shares a group of clinical features, mainly including polycystic kidney disease (PKD), defects in respiratory, retinal, neurological and hepatic, in addition to polydactyly, cranio-facial abnormalities, as well as obesity and diabetes caused by metabolic defects (Gerdes et al., 2009; Nigg and Raff, 2009; Hildebrandt et al., 2011; Waters and Beales, 2011; Valente et al., 2014; Ma et al., 2017).

Centriolar satellites are the membrane-free granules with a diameter ranging from 70 to 100 nm and move along microtubules in the vicinity of the centrosome (Balczon et al., 1994; Kubo et al., 1999; Dammermann and Merdes, 2002; Kubo and Tsukita, 2003). These structures, as dynamic protein complexes, are responsible for proteins trafficking from the cytoplasm toward the centrosome and ciliary complex, or vice versa (Barenz et al., 2011; Lopes et al., 2011; Tollenaere et al., 2015; Hori and Toda, 2017). Several ciliopathy-associated proteins, including Bardel Biedl syndrome 4 (BBS4), CEP290, pericentriolar material 1 (PCM1), and orofaciodigital syndrome 1 (OFD1), localize to centriolar satellites and are critical for cargos entry and/or exit from the primary cilium. BBS4 is a component of BBSome, which is a stable protein complex formed by eight conserved ciliary membrane-associated proteins (BBS1, BBS2, BBS4, BBS5, BBS7, BBS8, BBS9, and BBIP10) (Nachury et al., 2007; Nachury, 2008; Jin et al., 2010). PCM1 acts as a platform to recruit other satellite cargos and promote microtubule organization. OFD1, as a ciliary disease protein, also localizes to the distal end of mother centriole, which is converted into the basal body for the primary cilium formation upon cell division exit. This pool of OFD1 is responsible for the distal end decoration of centriole, IFT88 recruitment, and ciliogenesis (Ferrante et al., 2006; Singla et al., 2010). However, the population of OFD1 at centriolar satellites promotes the regular growth of the primary cilium via its degradation by autophagy under serum starvation conditions (Tang et al., 2013).

The transport protein particle (TRAPP) is a multi-subunit tethering protein complex that was initially identified in budding yeast (Sacher et al., 1998). The function of the yeast TRAPP has been well-documented, but that of mammalian TRAPP is still poorly understood. In yeast, the TRAPP complex has been found in three forms, TRAPPI, II and III, but only two forms, TRAPPII and III, have been identified in mammalian cells so far. The crystal structure of yeast TRAPPI revealed that TRAPPI as the core complex contains six subunits (Bet5p, Trs20p, Trs23p, Trs31p, Trs33p, as well as a couple of Bet3) and serves as the core for the assembly of TRAPPII and TRAPPIII. TRAPPI has not been identified as an independent complex in mammalian cells, but a six-subunit structure (TRAPPC1, TRAPPC2, TRAPPC4, TRAPPC5, TRPAAC6, and two copies of TRAPPC3) similar to yeast TRAPPI serves as the core for assembly of other specific subunits to form TRAPPII and TRAPPIII (Yu and Liang, 2012; Brunet and Sacher, 2014). In addition to the core, the mammalian TRAPPII also contains two specific subunits TRAPPC9 and TRAPPC10, homologues to yeast Trs120 and Trs130, and TRAPPIII contains TRAPPC8, TRAPPC13, homologues to yeast Trs85, and Trs65, as well as TRAPPC11 and TRAPPC12 not found in yeast (Yu and Liang, 2012; Bassik et al., 2013). TRAPPI complex, which acts as a tether with COPII vesicles derived from ER, mediates ER-to-Golgi transport (Sacher et al., 2001). TRAPPII mediates intra-Golgi transport, Golgi exit, endosome-to-Golgi traffic, as well as lipid droplet homeostasis (Brunet and Sacher, 2014; Li et al., 2017). Furthermore, TRAPPII interacts with Rabin8, which is a major GEF (guanine-nucleotide-exchange factor) for Rab8, to participate in ciliary vesicle formation during ciliogenesis (Westlake et al., 2011). TRAPPIII mainly functions in modulating COPII vesicle formation at the ER exit sites and regulates autophagy (Zhao et al., 2017). Recently, TRAPPC8, a subunit of TRAPPIII complex, was also found to serve in the trafficking of Rabin8 to the centrosome. However, it remains to be clarified whether the role of TRAPPC8 in ciliogenesis is related to its role as a TRAPPII protein.

Here, we investigate the different functions of two confirmed TRAPPIII components, TRAPPC8 and TRAPPC12, in the cilium formation using human retinal pigment epithelial (hTERT-RPE1) cells as a model. A tandem-affinity purification identified TRAPPC12-interacting protein, OFD1. We propose a novel model that explains how TRAPPIII regulates ciliogenesis via its interaction with a ciliopathy protein, OFD1.

## Materials and Methods

### Cell Culture

Mammalian cells used in this study were originally obtained from the American Type Culture Collection (ATCC, Manassas, Virginia). hTERT-RPE1 cells were cultured in Dulbecco's Modified Eagle's Medium/Nutrient Mixture F-12 medium (DMEM/F12) supplemented with 10% fetal bovine serum (FBS, Sigma, A6003). Other cell lines, Hela and HEK293T, were grown in DMEM (Invitrogen, 12800-017) containing 10% FBS and 1% penicillin-streptomycin solution. All cells were cultured at 37°C and 5% CO_2_ and detached by incubating in trypsin (Invitrogen, 12604-02) for 5 min. hTERT-RPE1 cells were serum-starved for 24–48 h to induce cilia formation in its described culture medium but with 0.5% FBS. After cilium formation upon serum starvation for 48 h, cilium disassembly was stimulated by re-introduction of serum to the cells for 24 h.

### Plasmids

Human OFD1 is subcloned into pMyc-CMV vector (Clontech). Mouse wild type and mutant forms of OFD1 (S75F, A80T, G139S, and S437R) were obtained from Addgene. Mouse TRAPPC8 and TRAPPC12 were obtained by our lab.

### RNAi

hTERT-RPE1 cells were transfected using LipofectamineR 3000 reagent with 80 nmol of siRNA targeting human TRAPPC8, human TRAPPC12, human OFD1, and human PCM1 mRNA. TRAPPC12: siGENOME human TRAPPC12 (Dharmacon, Cat. #M016861000005); TRAPPC8 siRNA (1): 5′-GAAGAUGGCCCUUGUACUAUU- 3′, TRAPPC8 siRNA (2): 5′-UAGUACAAGGGCCAUCUUCUU-3′ (designed by our lab); OFD1 siRNA (1): 5′-GCUCAUAGCUAUUAAUUCA-3′, OFD1 siRNA (2): 5′-GAUCGAUCGUUCUGUCAAU-3′ (Lopes et al., 2011); PCM1 siRNA: 5′-GGCUUUAA CUAAUUAUGGATT-3′ (Kim et al., 2008).

### Transfection

Transfections of DNA plasmids for gene expression in HEK293T cells and in Hela cells were done by PEI (Sigma, 408727) in a 3:1 plasmid concentration ratio, and JetPRIME transfection reagent (Polyplus, 114–15), respectively. Lipofectamine 3000 transfection reagent (ThermoFisher, L3000008) were used for plasmid or siRNA transfections in hTERT-RPE1 with a concentration of 80 nM siRNA (1 μl of Lipofectamine 3,000 per 1 ml medium) or 0.25 μg/ml plasmid (0.25 μl of Lipofectamine 3,000 per 1 ml medium).

### Immunoprecipitation

The cells were lysed in pre-chilled 800 μl of RIPA buffer (20 mM Tris pH 8.0, 100 mM NaCl and 0.1% NP-40) plus protease inhibitor cocktail and harvested by scraping. For complete lysis, tubes containing cell lysates were incubated on ice for 30 min, and vortexed at every 10 min. The lyophilized protein A-sepharose (sigma, P3391) was swollen in 1 × PBS for 5 min at room temperature, and then centrifuged at 2,000 rpm for 2 min at room temperature. After removal of the supernatants, the protein A beads were washed with 1 × PBS twice more. Finally, the protein A beads were mixed with the equal volume of 1 × PBS. 30 μl of the above beads in 1 × PBS was added into the lysate and 1 μg of antibody was also added to the reaction mixtures. The mixtures (lysate, beads and antibody) were incubated on a rotating wheel at 4°C overnight. Then the mixtures were centrifuged and the supernatants were removed. The beads were washed 3–5 times with RIPA buffer before sample buffer was added to the beads for immunoblotting analysis. Antibodies for blotting: mouse c-myc (9E10) (1:50, established by our lab), mouse anti-GAPDH (1:3000, EMD Millipore, CB1001), rabbit-GFP (FL) (1:500, Santa Cruz, sc-8334), rabbit-OFD1 (1:500, GeneTex, GTX110010), mouse-PCM1 (1:500, Santa Cruz, sc-398365), rabbit-PCM1 (1:500, Proteintech, 19856-1-AP), rabbit-TRAPPC8 (1:500, ThermoFisher, PA5-59429), rabbit-TRAPPC12 (1:2000, established by our lab), mouse-β-Actin (1:3000, ABclonal, AC-006), and horseradish peroxidase (HRP) conjugated secondary antibody were used at 1:5000 (Invitrogen).

### CRISPR/Cas9 System for Deletion of TRAPPC8

To knock out human TRAPPC8, three pairs of oligoes were designed by CRISPR gRNA Design tool-DNA2.0 (www.atum.bio/eCommerce/cas9/input). The oligos locate at 5′ upstream of a 3bp of NGG PAM sequence and shown as below:

C8KO-2 Forward: CACCGAATTAGGCAATTAAACGATC
C8KO-2 Reverse: AAACGATCGTTTAATTGCCTAATTC
C8KO-3 Forward: CACCGTAGAGCTCACCCACTTCAGT
C8KO-3 Reverse: AAACACTGAAGTGGGTGAGCTCTAC

Each pair of the above-mentioned annealed oligos was cloned into the digested guide RNA scaffold, lentiCRISPR v2 (Addgene, 52961). These plasmids were transfected into HEK293T cells, and the cells were selected by 2 μg/ml of puromycin for 24 h, and then by 1 μg/ml of puromycin for additional 48 h. The efficiency of the knockout was analyzed by PCR and western blot. The potential cells were seeded into 96-well for selection of a single cell. Overall, we got two populations of single cells with TRAPPC8 deletion from 35 samples. Procedures for getting TRAPPC12 deletion cells have been described previously (Zhao et al., 2017).

### Immunofluorescence and Microscopy

Cells were grown on 12 mm glass coverslips for 24 h prior to the treatments (siRNA or plasmid transfection and/or starvation) and washed three times with 1 × PBS before fixation. Fixation was carried out with 3.7% formaldehyde was used for 15 min at room temperature or with 100% methanol (−20°C) on ice for 5 min. Then, the cells fixed with formaldehyde (PFA) were permeabilized with 0.1% Triton X-100 in 1 × PBS for 5 min at room temperature, before blocking with blocking buffer (1 × PBS containing 1% BSA) for 1 h. Then, primary antibodies were applied to the samples for 2 h at room temperature. The cells were washed three times with 1 × PBS, and then incubated with appropriate secondary antibodies for 1 h at room temperature. DAPI was used to counterstain for nucleus. Images were captured by an FV1200 Olympus inverted confocal microscopy equipped SIM scanner, with 100X objective. Same exposures were always used in imaging for the comparable samples. Antibodies for immunofluorescence: rabbit-ARL13B (1:300, Proteintech, 17711-1-AP), mouse-ERGIC53 (1:3000), a gift from Prof. Hans-Peter Hauri, mouse-GM130 (1:100, BD, 610823), rabbit-OFD1 (1:100, Novus, NBP1-89355), mouse-PCM1 (1:300, Santa Cruz, sc-398365), rabbit-PCM1 (1:300, Proteintech, 19856-1-AP), rabbit-Sec31A (H-143) (Santa Cruz, sc-98523), rabbit-TRAPPC8 (1:100, Thermo Fisher PA5-59429), TRAPPC12 (1:50, established by our lab). The antibodies were diluted in blocking buffer.

### Ciliary Length Measurements

Cilium length was measured by Image J. The length of the scale bar (10 μm) was set as the scale distance in pixels. The segmented lines along cilia were drawn, and their length was measured to represent cilium length.

### Quantification and Statistical Analysis

For fluorescent quantitative analysis, OFD1 and PCM1 puncta in hTERT-RPE1 cells were carried out by measuring the intensities of fluorescence in a respective 4 and 8 μm^2^ circular areas around the centrosome. Statistical analyses were carried out with Prism 6 software (GraphPad). The data were expressed as mean ± SEM (Standard Error of Mean) or mean± SD (Standard Deviation). The differences between two number groups were compared by two tailed unpaired *T*-test (*p* < 0.05), while within multiple groups were analyzed by no matching or pairing one-way ANOVA followed by bonferroni' multiple comparison test (*p* < 0.05).

## Results

### TRAPPIII Localizes to the Basal Body

**Figure 1:**
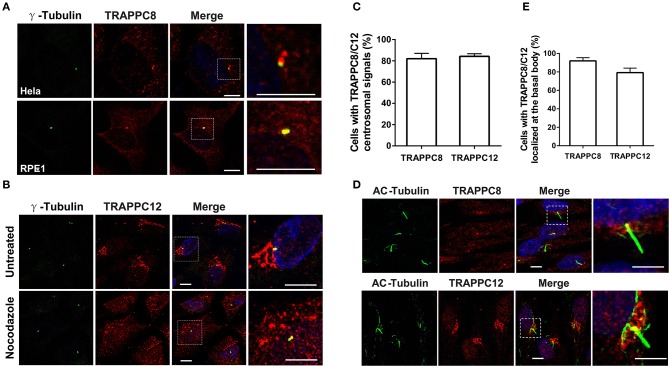
TRAPPC8 and TRAPPC12 are localized to basal bodies and centrosomes. **(A)** TRAPPC8 was co-localized with centrosome marker γ-tubulin in Hela and hTERT-RPE1 cells. **(B)** TRAPPC12 was co-localized with γ-tubulin in hTERT-RPE1 cells that were prior treated with or without nocodazole for 1 h. **(C)** Quantifications of data shown in **(A,B)**. Percentage of cells with TRAPPC8 or TRAPPC12 centrosomal signals were scored TRAPPC8, *n* = 82; TRAPPC12, *n* = 72. Mean ± SEM. **(D)** TRAPPC8 and TRAPPC12 were stained with axoneme marker acetylated α-Tubulin (AC-tubulin) in hTERT-RPE1 cells after the cells were induced to form cilia with serum starvation for 24 h. observe **(E)** Percentage of cells with TRAPPC8 or TRAPPC12 signals localized at the basal body. TRAPPC8, *n* = 108; TRAPPC12, *n* = 96. Mean ± SEM. Scale bar, 10 μm. Similar results were observed in three independent experiments.

TRAPPC8 is a major subunit of TRAPPIII, and therefore, knowing its subcellular location is important for understanding its function. We investigated the localization of TRAPPC8 in ciliated cells and cancer cells with antibodies to TRAPPC8 and several intracellular organelle markers. We chose hTERT-RPE1, Hela, and HEK293T cells because hTERT-RPE1 is the human retinal pigment epithelial cell line immortalized with telomerase reverse transcriptase. In this cell line, the mother centriole projects a single primary cilium under the clearly defined condition such as serum starvation, making it very easy to score the efficiency for cilliogenesis. Second, we have been studying membrane trafficking using Hela and HEK293T cells (Zong et al., 2012; Gan et al., 2017; Li et al., 2017; Zhao et al., 2017; Satoh et al., 2019). These cells are easy to grow and show no obvious abnormalities in general secretion. Interestingly, when cells were fixed by methanol, the signals of TRAPPC8 were associated with centrosomal marker, γ-Tubulin in both ciliated hTERT-RPE1 cells and Hela cells (Figures 1A,C). In hTERT-RPE1 cells under serum starvation, TRAPPC8 signal was localized to the basal body, which is the base of the primary cilium (Figures 1D,E). In contrast, when Hela cells fixed with paraformaldehyde (PFA) and permeabilized with Triton X-100, TRAPPC8 signal presented fluorescent puncta co-localized with GM130, Sec31A, and ERGIC53 (Figure S1A). In hTERT-RPE1 cells fixed with PFA, TRAPPC8 did not show labeling in ER exit sites or ERGIC, possibly due to its weak endogenous signal. To further examine the localization of TRAPPC8 to these organelles, we also investigated the distribution of TRAPPC8 upon treatment with an antineoplastic agent, nocodazole. Nocodazole rapidly depolymerizes microtubules and causes the membrane organelles to fragment into small, scattered punctate structures (Rogalski et al., 1984). One hour after application of nocodazole, TRAPPC8 signal was mostly associated with fragmented Sec31A and ERGIC53, but poorly with GM130, as shown in Figure S1A, confirming TRAPPC8 in ER exit sites and the intermediate compartments.

It was reported that TRAPPC12 also localized to the ER exit sites and ERGIC in cancer cells (Zhao et al., 2017). The localization of TRAPPC12 in cells with primary cilia is, however, not determined. To investigate its localization in ciliated hTERT-RPE1 cells, we performed TRAPPC12 immunofluorescence after methanol fixation. TRAPPC12 signal was present in large punctate structure mostly on ER exit sites and ERGIC, but poorly on Golgi apparatus (Figure S1B), as the previous report in cancer cells. Additionally, another pool of TRAPPC12 signal was found on the centrosome and the base of the primary cilium (Figures 1B–E). To confirm that TRAPPC12 is also a centrosome/basal body-associated protein, we investigated its distribution with the treatment of nocodazole. TRAPPC12 was observed to be on small, discrete puncta dispersed in the cytoplasm, and one of puncta was localized to the centrosome in almost every cell (Figures 1B,C). Taken together, these data reveal that TRAPPIII localizes to the basal body, as well as the early secretory pathway.

### TRAPPC8 and TRAPPC12 Play Different Roles in Ciliary Length

**Figure 2:**
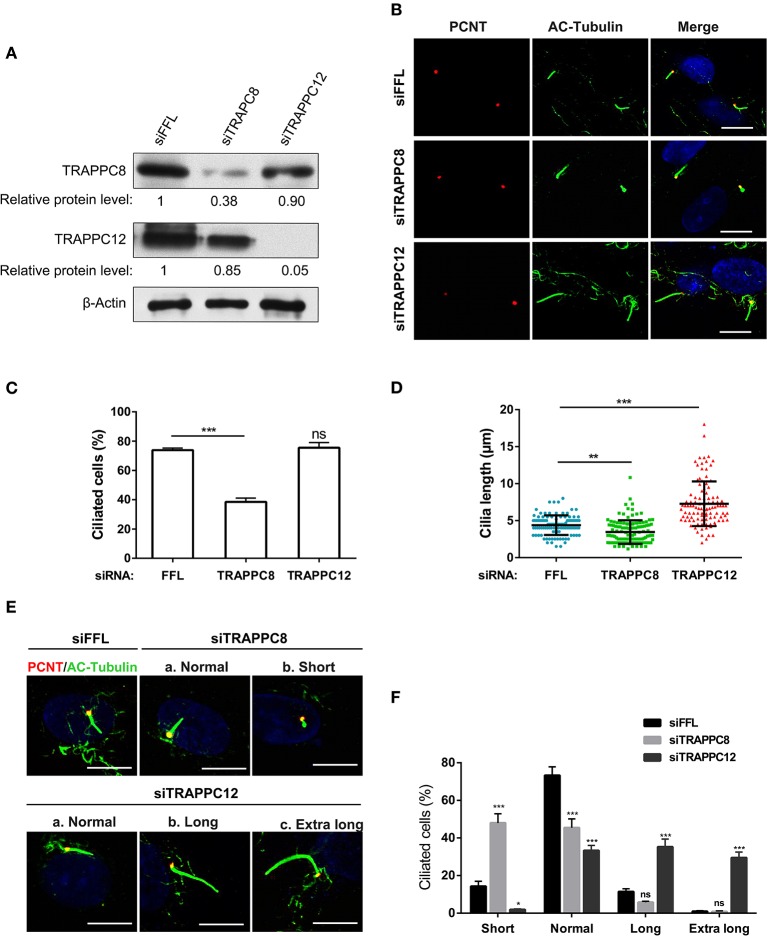
TRAPPC8 and TRAPPC12 depletions change ciliary length. **(A)** hTERT-RPE1 cells were transfected with control (siFFL), TRAPPC8 siRNA (siTRAPPC8) or TRAPPC12 siRNA (siTRAPPC12) for 72 h. FFL siRNA targets the firefly luciferase gene sequence, which is not present in human genome, and therefore, serves as non-targeting siRNA control. The expression level of the indicated proteins was analyzed from whole cell lysates by immunoblotting using antibodies against TRAPPC8, and TRAPPC12. β-Actin served as a loading control. **(B)** hTERT-RPE1 cells depleted TRAPPC8 or TRAPPC12 were induced with primary cilum formation. The approximate positions of the centrosome were indicated by PCNT (pericentrin) staining and primary cilia were indicated by AC-tubulin. **(C,D)** Percentage of cells with primary cilia and quantification of ciliary length in ciliated cells. Cilium length was measure by imageJ software and based on the scale bar length at 10 μm to set measurement scale. siFFL, *n* = 105; siTRAPPC8, *n* = 121; siTRAPPC12, *n* = 105. Mean ± SEM, *p < 0.05;**p < 0.01; ***p < 0.001, no matching or pairing one-way ANOVA. **(E)** TRAPPC8 depletion caused short primary cilia, while TRAPPC12 depletion caused aberrantly long primary cilia. **(F)** Percentage of cells with short cilia (0–3 μm); with normal cilia (3–6 μm); with long cilia (6–8 μm); with extra long cilia (>8 μm). siFFL, *n* = 168; siTRAPPC8, *n* = 185; siTRAPPC12, *n* = 165. no matching or pairing one-way ANOVA. Similar results were observed in three independent experiments.

The basal body localization of TRAPPIII makes us wonder whether TRAPPIII has a function in ciliogenesis. To investigate the role of TRAPPIII in ciliogenesis, we analyzed the primary cilium formation in serum-starved hTERT-RPE1 cells depleted for TRAPPC8 or TRAPPC12 using small interfering RNA (siRNA) (Figure 2A). Similar to the previous report (Schou et al., 2014), TRAPPC8 depletion by siRNA resulted in a more than 35% decrease in cilium assembly compared with controls (Figures 2B,C). Moreover, for the cells that did assemble primary cilia, half of them displayed aberrant cilia with the shorter axoneme and bulbous tips (~2.0 μm in ciliary length compared to 4.5 μm for controls) (Figures 2C–F). In contrast, depletion of TRAPPC12 exhibited similar ciliogenesis efficiency as control cells (Figure 2C). We surprised to find that a significant portion of TRAPPC12-depleted cells formed unusually long primary cilia, measuring up to 18 μm in length with a median ciliary length of 7.28 μm compared to 4.5 μm for controls (Figures 2C,D,F). There was no observable abnormality in the TRAPPC12 depleted cilia other than increase in length (Figure 2E). These data imply a possible regulatory role of TRAPPC12 in ciliary elongation and a requirement of TRAPPC8 in normal cilium formation.

**Figure 3:**
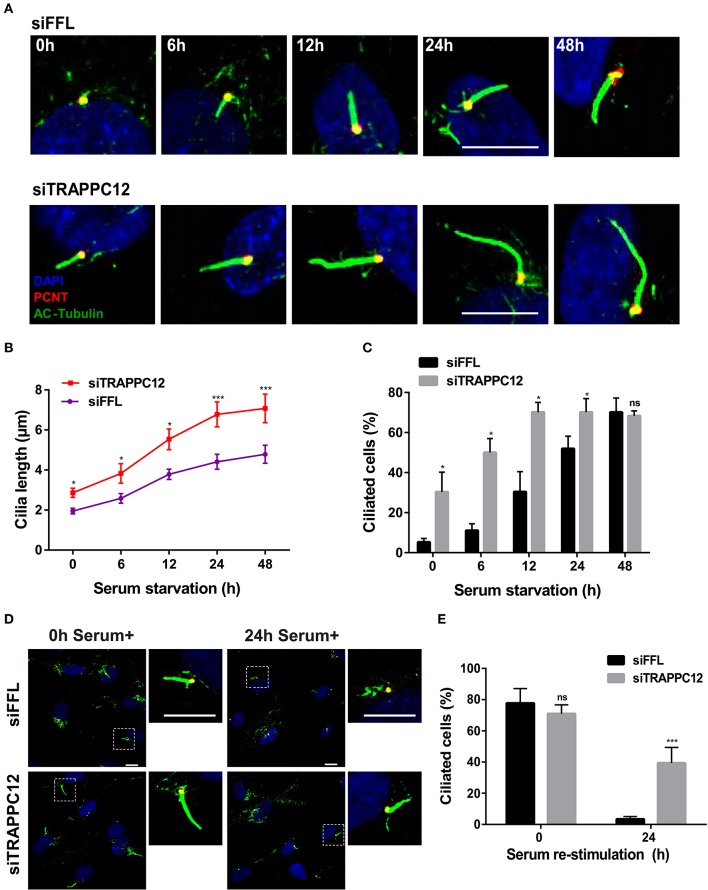
TRAPPC12 regulates cilia disassembly. **(A)** Time course of ciliogenesis induced by serum starvation. siFFL and siTRAPPC12 transfected hTERT-RPE1 cells were serum starved at the five indicated time points. **(B,C)** Statistical quantifcations of cilia length. Number of cilia measured at 0 h: siFFL, *n* = 13; siTRAPPC12, *n* = 27; 6 h: siFFL, *n* = 17; siTRAPPC12, *n* = 40; 12 h: siFFL, *n* = 46; siTRAPPC12, *n* = 58; 24 h: siFFL, *n* = 100; siTRAPPC12, *n* = 120; 48 h: siFFL, *n* = 100; siTRAPPC12, *n* = 100. Mean ± SEM for average ciliary length, ***p < 0.001; Mean ± SD for ciliated cells, *p < 0.05, two tailed unpaired *T*-test for two groups of each time point. **(D)** siFFL and siTRAPPC12 hTERT-RPE1 cells were serum starved for 48 h (0 h serum^+^) and re-simulated by serum (10%) for 24 h (24 h serum^+^). **(E)** Quantification of ciliated cells with mean ± SD, ***p < 0.001; two tailed unpaired *T*-test for two groups of each time point. Scale bar, 10 μm. Similar results were observed in three independent experiments.

The formation of these aberrant cilia in hTERT-RPE1 cells was confirmed by staining for ciliary membrane using ARL13B, which specifically localizes at the cilium membrane (Cantagrel et al., 2008) (Figure S2). To further examine this finding, we performed a comparative study of ciliation frequency at several time points following serum starvation. As shown in Figures 3A–C, TRAPPC12 depletion led to the earlier occurrence of the cilium formation than control at all time points investigated, and TRAPPC12-depleted cells contained longer primary cilia than control cells. Remarkably, 30.3% cells were found ciliated after TRAPPC12 depletion even when the cells were cultured in the presence of serum (Figure 3C). An abnormally elongated cilium on mammalian cells is usually involved in the delayed or failed ciliary resorption. We speculated that the long cilium in TRAPPC12-depleted cells be caused by the defective cilium disassembly. We arrested the control and TRAPPC12-depleted cells in G0 phase of the cell cycle by serum starvation for 48 h, and during this time, cilia were formed. Then, we stimulated cilium disassembly by re-introduction of serum to the cells for 24 h and estimated the efficiency of cilium disassembly. Indeed, ciliary resorption was not completed in TRAPPC12-depleted cells: 39.4% still had cilia, compared to 3.4% in siFFL-depleted cells (Figures 3D,E). Together, these data indicate that long cilium resulted from a defect in ciliary disassembly caused by TRAPPC12 depletion.

### TRAPPIII Interacts With OFD1

**Figure 4:**
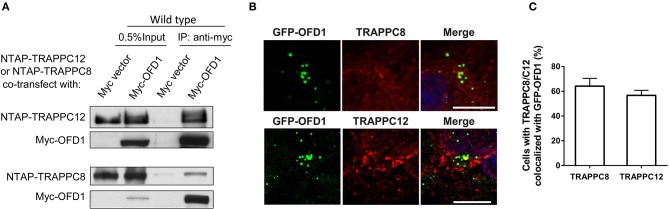
TRAPPIII interacts with OFD1. **(A)** Myc-tagged OFD1 (Myc-OFD1) and NTAP-tagged TRAPPC8 (NTAP-TRAPPC8) or TRAPPC12 (NTAP-TRAPPC12) were co-transfected into the HEK293T cells, and then cell lysates were subjected to immunoprecipitation with an anti-Myc antibody. Approximately 30% of immunoprecipitants were loaded on SDS PAGE **(B)** hTERT-RPE1 cells were transfected with GFP-tagged OFD1 and stained for TRAPPC8 and TRAPPC12 with antibodies to determine the colocalization of TRAPPC8 or TRAPPC12 and OFD1. **(C)** Percentage of cells with TRAPPC8 or TRAPPC12 colocalized with GFP-OFD1. TRAPPC8, *n* = 34; TRAPPC12, *n* = 38. Mean ± SEM. Scale bar, 10 μm. Similar results were observed in three independent experiments.

**Figure 5:**
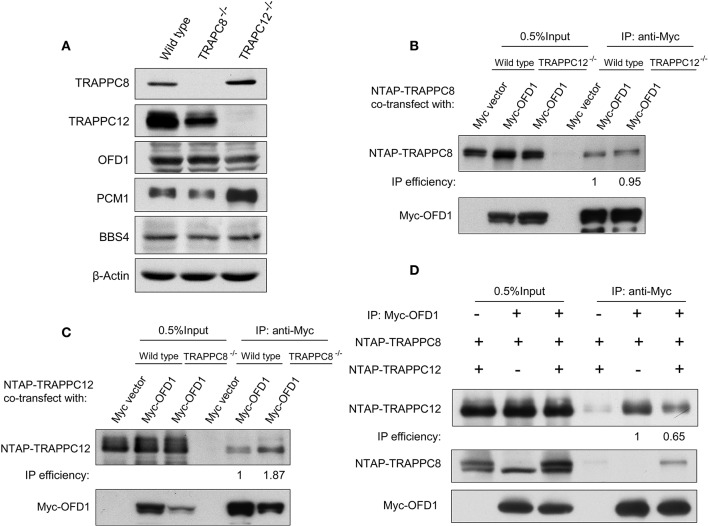
TRAPPC8 binds OFD1 and inhibits TRAPPC12-OFD1 interactions. **(A)** Immunoblotting analysis of TRAPPC8, TRAPPC12, OFD1, PCM1, and BBS4 proteins in wildtype control, TRAPPC8- or TRAPPC12-deleted HEK293T cells. β-Actin served as a loading control. **(B)** The interaction between Myc-tagged OFD1 (Myc-OFD1) and NTAP-tagged TRAPPC8 (NTAP-TRAPPC8) were determined in the wild type or TRAPPC12^−/−^ HEK293T cells by co-IP experiment. Approximately 30% of immunoprecipitants were loaded for analysis. **(C)** The interaction between Myc-tagged OFD1 (Myc-OFD1) and NTAP-tagged TRAPPC12 (NTAP-TRAPPC12) were determined in the wild type or TRAPPC8^−/−^ HEK293T cells by co-IP experiment. Approximately 30% of immunoprecipitants were loaded for analysis. **(D)** NTAP-TRAPPC8 and NTAP-TRAPPC12 were co-expressed with Myc-OFD1 to allow competition of binding. Co-IP experiment was performed to pull down Myc-OFD1 and the extent of binding by TRAPPC8 or TRAPPC12 was investigated by immunoblotting. IP efficiency is calculated as the ratio of co-immunoprecipitated OFD1 proteins in TRAPPC8 and TRAPPC12 IP samples. Similar results were observed in three independent experiments.

We have previously established the tandem affinity purification (TAP) to identify TRAPPIII-interacting proteins (Figure S3). TRAPPC12-OFD1 interaction caught our attention because OFD1 was reported to regulate ciliogenesis and ciliary length. To confirm the interaction between TRAPPC12 and OFD1, co-immunoprecipitation (co-IP) experiment was carried out with both NTAP-TRAPPC12/TRAPPC8 (NTAP-TRAPPC12/NTAP-TRAPPC8) and Myc tagged OFD1 (Myc-OFD1) co-transfected into HEK293T cells. We found that OFD1 physically interacted with both TRAPPC8 and TRAPPC12 (Figure 4A). Furthermore, overexpression of GFP-tagged OFD1 (GFP-OFD1) revealed colocalization with TRAPPC8 and TRAPPC12 at the centrosome in hTERT- RPE1 cells (Figures 4B,C). We further investigated how TRAPPC8 or TRAPPC12 interact with OFD1 using HEK293T cell line deleted with each of these TRAPPIII subunits (Figure 5A). We found that the interaction between OFD1 and TRAPPC8 was the same in wild type and TRAPPC12^−/−^ HEK293T cells (Figure 5B), but the interaction between TRAPPC12 and OFD1 was enhanced in TRAPPC8^−/−^ HEK293T cells (Figure 5C). When we co-expressed all three proteins and tested for their interactions, we confirmed that the presence of TRAPPC8 inhibited the interaction between TRAPPC12 and OFD1 (Figure 5D).

**Figure 6:**
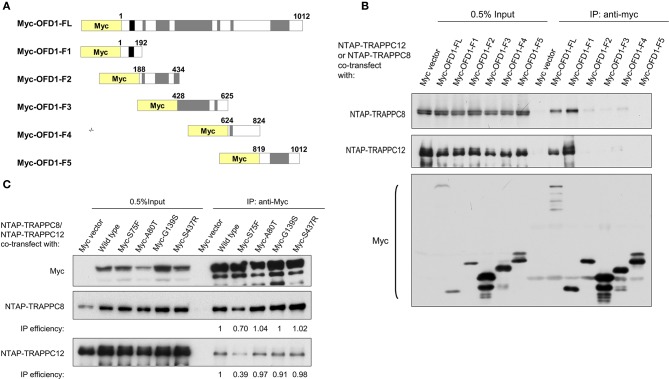
TRAPPIII interacts with the LisH domain of OFD1. **(A)** Schematic diagram of the indicated full-length OFD1 (FL) and its truncation constructs. The LisH (black), Coiled-coil (grey) and the numbers of amino acid residues in each constructs are indicated. **(B)** Lysates from HEK293T cells co-transfected with various OFD1 deletion constructs and NTAP-TRAPPC8 or NTAP-TRAPPC12 were subjected to immunoprecipitation and immunoblotted for Myc and TRAPPC8 and TRAPPC12. **(C)** OFD1 mutant S75F reduces the interaction with TRAPPC8 and TRAPPC12. The indicated OFD1 mutants were co-expressed with TRAPPC8 or TRAPPC12 and the strength of their interactions were determined by co-IP experiment. IP efficiency is calculated as the ratio of immunoprecipitated TRAPPC8/OFD1 or TRAPPC12/OFD1. Similar results were observed in three independent experiments.

To determine the region(s) of OFD1 responsible for TRAPPIII binding, we performed domain mapping experiment. OFD1 consists of a short N-terminal domain, Lis 1 homology (LisH) required for microtubule dynamics and its centriolar satellite localization, and six much longer coiled-coil domains that are responsible for its centrosome localization (Romio et al., 2004) (Figure 6A). The indicated Myc-tagged OFD1 truncation constructs were transfected into HEK293T cells, and co-IP experiment was performed. The N-terminal region (residues 1–192), including LisH motif, interacted with TRAPPC8 even stronger than full-length OFD1 (Figure 6B). Similarly, TRAPPC12 also interacted with the N-terminal region (LisH motif) and the C-terminal coiled-coil domains had no significant binding with TRAPPC12 (Figure 6B). We, therefore, concluded that OFD1 interacts with TRAPPC8 and TRAPPC12 via its N-terminal region including LisH motif. The significance of these findings is highlighted by the fact that several mutations observed in human OFD1 patients lie within the regions required for its TRAPPIII interaction. These mutations, S75F, A80T, G139S, and S437R, are four main disease-associated missense mutations found in patients with disease of OFD1. S75F and A80T lie within the LisH motif, and G139S affects intervening conserved amino acids, and S437R has an effect on the second coiled-coil motif (Ferrante et al., 2001; Rakkolainen et al., 2002; Romio et al., 2004; Thauvin-Robinet et al., 2006). In Figure 6C, the binding of OFD1 protein containing one of these disease-associated mutations with TRAPPIII components was examined. S75F, but not other indicated mutations reduced the interaction between OFD1 and TRAPPC8 and TRAPPC12. S75F mutation resides in the LisH domain, which is required for the localization of OFD1 at centriolar satellites, and therefore, we suspect that TRAPPIII may regulate ciliogenesis via controlling the localization of OFD1 at centriolar satellites.

### TRAPPIII Components Regulate the Distribution of OFD1

**Figure 7:**
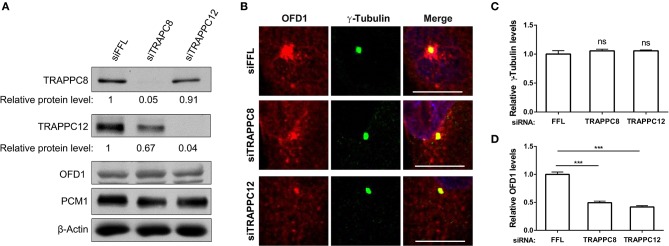
TRAPPIII depletion reduces the amount of OFD1 at centriolar satellites. **(A)** The efficiency of depletion for TRAPPC8 and TRAPPC12. The expression level of the indicated proteins was analyzed from whole cell lysates by immunoblotting using antibodies against TRAPPC8, TRAPPC12, OFD1, and PCM1. β-Actin served as a loading control. **(B)** Depletion of TRAPPC8 or TRAPPC12 reduced OFD1 puncta. Endogenous OFD1 and the centrosome were detected with OFD1 antibody and the centrosome marker γ-Tubulin in hTERT-RPE1 cells. **(C)** Quantitative analysis of γ-Tubulin puncta. **(D)** Quantitative analysis of OFD1 puncta was carried out by measuring the intensities of fluorescence in a 4 μm^2^ circular area around the centrosome by imageJ. siFFL, *n* = 100; siTRAPPC8, *n* = 100; siTRAPPC12, *n* = 80. Mean ± SEM, ***p < 0.001, no matching or pairing one-way ANOVA. Scale bar, 10 μm. Similar results were observed in three independent experiments.

To address whether OFD1 localization was affected by TRAPPIII, we used siRNA to deplete each of these TRAPPIII components, TRAPPC8 and TRAPPC12 in hTERT-RPE1 cells. The efficiency of TRAPPC8 and TRAPPC12 depletion was determined (Figure 7A). TRAPPC8 or TRAPPC12 depletion did not alter the abundance of the centrosome marker, γ-Tubulin (Figures 7B,C). However, depleting TRAPPC8 resulted in a significant reduction in the number of OFD1 puncta in the vicinity of the centrosome, and this effect was even more dramatic in TRAPPC12 depletion (Figures 7B,D). The pericentriolar signals of OFD1 almost completely disappeared in TRAPPC12-depleted cells, but the pool of OFD1 at centrosome was retained (Figures 7B,D).

We were aware that the secretory pathway was also affected by TRAPPC8 or TRAPPC12 depletion, as such change had already been reported in our previous publication. As shown in Figure S4A, both TRAPPC8 and TRAPPC12 depletions disrupted the Golgi morphology in hTERT-RPE1 cells, similar to what had previously been shown in Hela cells (Zhao et al., 2017). In addition, the ER related organelle markers, ERGIC53 and Sec31A were also fragmented into small puncta (Figure S4A). In fact, we were able to analyze the effect on OFD1 using cell with dispersed Golgi as indicator of TRAPPC8 or TRAPPC12 depletion. To determine the observed change of OFD1 was not caused by the Golgi fragmentation indirectly, we tested the distribution of OFD1 in hTERT-RPE1 cells with the treatment of Brefeldin A (BFA). BFA is a fungal metabolite which inhibits ER-to-Golgi vesicle trafficking and thereby causes the fragmentation of the Golgi apparatus. As shown in Figures S4C,D, BFA treatment led to Golgi dispersal but the abundance of OFD1 was not altered. Hence, depletion of TRAPPIII components led to a decrease in the satellite OFD1 (Figure S4B). We confirmed that even though depletion of TRAPPIII decreased the OFD1 signals at centriolar satellites, the overall OFD1 protein levels indicated by immunoblot analysis of whole cell lysates were not altered in both depleted cells (Figure 7A). This observation suggests that depletion of TRAPPIII components likely results in a redistribution of OFD1 from centriolar satellites to cytoplasmic satellites, suggesting TRAPPIII is required for the recruitment of OFD1 to centriolar satellites.

### TRAPPC12 and TRAPPC8 Depletion Disperses Centriolar Satellites

**Figure 8:**
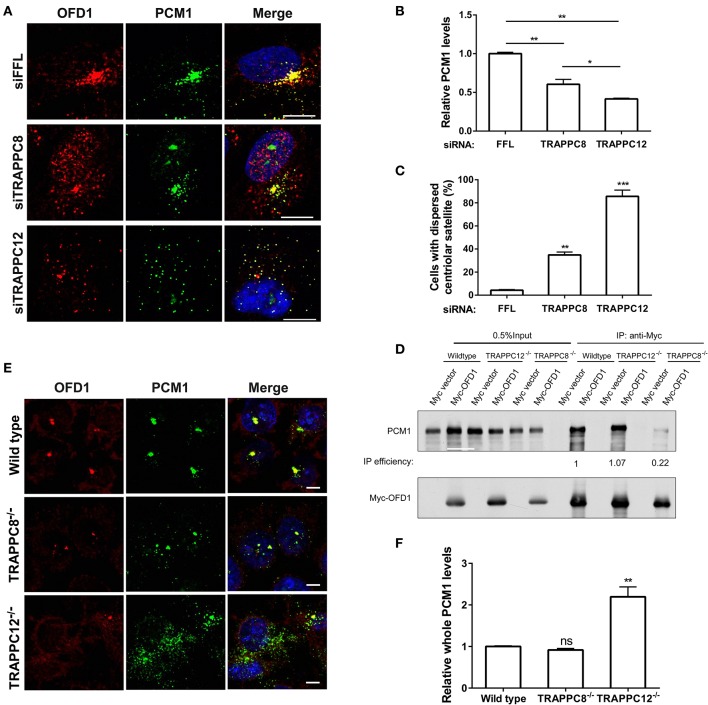
TRAPPIII regulates the assembly of centriolar satellites. **(A)** Depletion of TRAPPC8 reduced the PCM1 signals and colocalization with OFD1 and depletion of TRAPPC12 dispersed PCM1 signals in hTERT-RPE1 cells. Enhanced the exposure for OFD1 and PCM1 signals in hTERT-RPE1 cells with the depletion of TRAPPC8 or TRAPPC12. **(B,C)** Quantitative analysis of PCM1 puncta and percentage of cells with dispersed centriolar satellites. The intensities of fluorescence were measured in the 8 μm^2^ circular area around the centrosome by image J. siFFL, *n* = 50; siTRAPPC8, *n* = 48; siTRAPPC12, *n* = 50. Mean ± SEM, *p < 0.05; **p < 0.01; ***p < 0.001, no matching or pairing one-way ANOVA. **(D)** Myc-OFD1 was transfected in HEK293T wild type, TRAPPC12^−/−^, and TRAPPC8^−/−^ cells, and co-IP was performed with an anti-Myc antibody and the presence of endogenous PCM1 in the immunoprecipitants were detected by western blotting with anti-PCM1 antibody. IP efficiency is calculated as the ratio of immunoprecipitated PCM1/OFD1. **(E)** hTRAPPC12 knockout (TRAPPC12^−/−^) dispersed PCM1 signals in 293T cells. **(F)** Quantitative analysis of endogenous PCM1 puncta, the intensities of fluorescence were measured in whole cell by image J. Wild type, *n* = 40; TRAPPC8^−/−^, *n* = 48; TRAPPC12^−/−^, *n* = 50. Mean ± SEM, **p < 0.01, no matching or pairing one-way ANOVA. Scale bar, 10 μm. Similar results were observed in three independent experiments.

PCM1 is the platform of centriolar satellites, and thus its localization reflects the organization of centriolar satellites (Tollenaere et al., 2015). Depletion of PCM1 reduced the satellite localization of OFD1 and vice versa (Lopes et al., 2011) (Figures S5A–C). Based on this role, we investigated the relationship between PCM1 and OFD1 after we depleted TRAPPC8 or TRAPPC12 in hTERT-RPE1 cells. OFD1 signals at the centriolar satellites were dispersed in depletion of TRAPPC8 and TRAPPC12 but its signal at the centrosome remained intact (Figures 8A–C). Strikingly, TRAPPC8 depletion led to a decrease in the number fluorescence puncta of PCM1 at centriolar satellites. These puncta were poorly co-localized with the OFD1 signal (Figure 8A). This result was confirmed by the lack of physical interaction between the two proteins in TRAPPC8^−/−^ HEK293T cells, whereas this interaction was not affected in TRAPPC12^−/−^ HEK293T cells (Figure 8D). To further confirm these IP findings, we also analyzed OFD1 and PCM1 distribution in TRAPPC8^−/−^ and TRAPPC12^−/−^ 293T cells. For any reasons that still await further investigation, we found that the total PCM1 fluorescence was increased in TRAPPC12^−/−^ cells (Figures 8E,F). Immunoblotting of the lysate isolated from TRAPPC12^−/−^ cell lines showed increased PCM1 protein level (Figure 5A).

These results strongly suggest TRAPPC12 is required for PCM1 to be associated with the centriolar satellite, so that it serves as a platform and recruits proteins such as OFD1 and pericentrin (PCNT). PCNT puncta was dramatically reduced upon depletion of PCM1 (Figures S6A,B), suggesting the delivery or the association of PCNT with centrosome is dependent on PCM1. However, TRAPPC12 depletion did not affect PCNT at the centrosome (Figures S6C,D). This suggests that the functional integrity of the centriolar satellites was not compromised by TRAPPC12 depletion and reduced association of PCNT at centrosome is not necessarily dependent on the status of PCM1. Together, these results are consistent with the hypothesis that TRAPPC12 plays a significant role in the structural integrity of centriolar satellites, the function of which remains largely unaltered in TRAPPC12 depletion. This notion is supported by our observation that BBSome was transported onto the cilium of TRAPPC12 depleted cells. BBS9 was transported to the cilium of TRAPPC12 depleted cells (Figures S6E,F). This suggests that molecular trafficking still could occur in spite of the mislocalization of PCM1.

## Discussion

We identified both TRAPPC8 and TRAPPC12 interact with a ciliopathy-related protein OFD1. The interaction of OFD1 with TRAPPC12 is increased, and with PCM1 is strongly reduced by the depletion of TRAPPC8. This observation confirms the requirement of OFD1-PCM1 interaction in ciliary growth because TRAPPC8 depletion reduced the number of ciliated cells and ciliary length. At present, however, we cannot rule out the possibility that the increased interaction between TRAPPC12 and OFD1 in the absence of TRAPPC8 might have been solely responsible for the defects in ciliogenesis in the TRAPPC8 depletion. In contrast, TRAPPC12 depletion does not disrupt the interaction between OFD1 and PCM1 nor change that between OFD1-TRAPPC8, but TRAPPC12 depletion caused PCM1 dispersal. We think that even though the subcellular location of centriolar satellites becomes fragmented in TRAPPC12 depleted cells, the function of this organelle remains intact. In particular, BBSome trafficking does not appear to be affected in the TRAPPC12 depleted cells. We also found BBS4 was completely mislocalized in TRAPPC12^−/−^ cells (Figure S7). To our surprise, BBSome trafficking is functionally intact, even enough to generate abnormally long cilia even the centriolar satellite is dispersed. This observation is somewhat reminiscent of the role of Giantin on Golgi organization. Giantin serves as Golgi tether and its depletion causes fragmentation of the Golgi apparatus into mini-stacks. Though structurally compromised, Giantin-depleted cells have increased anterograde transport (Koreishi et al., 2013). Based on such similarity, we suspect the function of TRAPPIII at the centriolar satellites could be a tether, bringing together OFD1 and PCM1.

**Figure 9:**
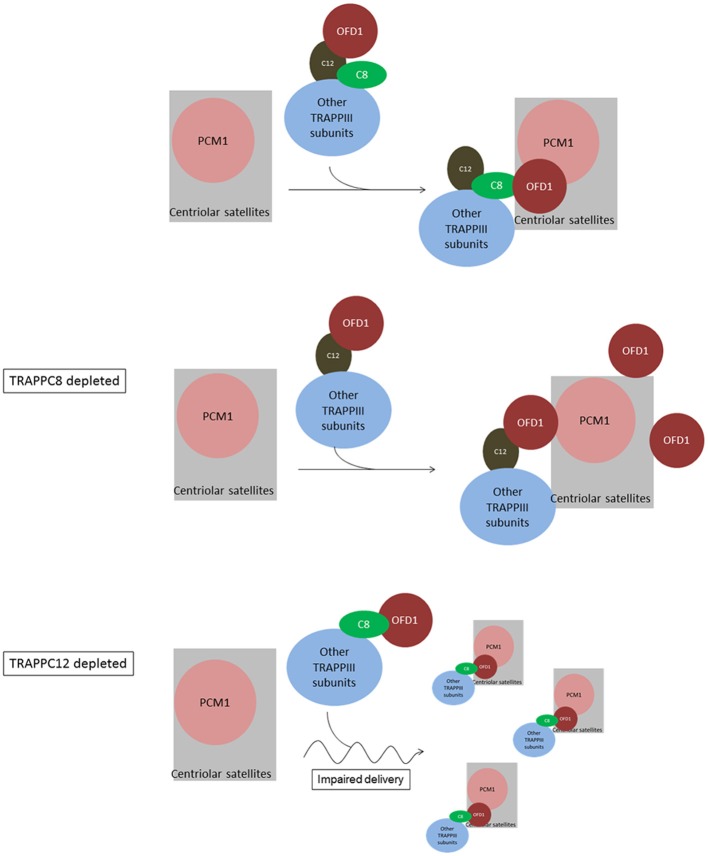
Summary and working model of how TRAPPIII functions in the assembly of centriolar satellites and ciliogenesis. We hypothesize sequential interactions between OFD1 and TRAPPC12 and TRAPPC8. TRAPPC12 serves a chaperonin-like function and binds to OFD1, but this interaction is out-competed by TRAPPC8 within the TRAPPIII complex. TRAPPC8 has the ability to properly position OFD1 to the centriolar satellites so that OFD1 can interact with PCM1. In TRAPPC8 depleted cells, the TRAPPIII complex without TRAPPC8 is able to bind to OFD1 via TRAPPC12, but positioning of OFD1 to the centriolar satellites, and hence the interaction with PCM1, is defective. On the other hand, OFD1 positioning to the centriolar satellites is intact in TRAPPC12 depletion. TRAPPC12 also has a function in help delivery of BBS4 or associated cargo proteins to the centriolar satellites. Without TRAPPC12, impaired BBS4/cargo delivery disperses the centriolar satellites.

Based on the data at hand, we propose a model to depict the role of TRAPPIII in the assembly of centriolar satellites (Figure 9). TRAPPC8 may regulate the stable association of OFD1 with centriolar satellites, and TRAPPC12 may regulate the trafficking of OFD1 from cytosol to centriolar satellites. When TRAPPC8 is depleted, more OFD1 interacts with TRAPPC12 (Figure 5B), and less interacts with PCM1 to assemble centriolar satellites. However, when TRAPPC12 was depleted, OFD1 could not be carried to the sites of centriolar satellites that contain PCM1, and therefore, the integrity of centriolar satellites is compromised and PCM1 is dispersed throughout the cytoplasm. The domain mapping experiments also suggest these two TRAPPIII components interact with the same domain on OFD1 and TRAPPC8 inhibits the interaction between TRAPPC12 and OFD1 but not vice versa. This observation supports the role of TRAPPC12 being a chaperonin for OFD1 and TRAPPC8-OFD1 interaction being more stable. Distinct roles of TRAPPC8 and TRAPPC12 have been reported previously. They have different roles in autophagy even though they are members of the same protein complex (Behrends et al., 2010; Lamb et al., 2016). TRAPPC8 depletion results in defects in the early stage of autophagy. Several studies have shown that the effective degradation of satellite OFD1 through autophagy is essential for ciliary elongation (Tang et al., 2013; Gabriel et al., 2016). Even though depletion of TRAPPC8 reduces the association of OFD1 with PCM1, reduced autophagy allows the remaining OFD1 be located at centriolar satellites in TRAPPC8-depleted cells upon serum starvation. This may explain why TRAPPC8-depleted cells displayed short cilia. The opposing roles of TRAPPC8 and TRAPPC12 in the control of ciliary length are probably related to their functions in the assembly of centriolar satellites. Ciliary formation and elongation are simultaneously compromised by the reduced autophagy associated with TRAPPC8 depletion. However, depletion of TRAPPC12 leads to the mislocalization of OFD1, and this complete loss of OFD1 from centriolar satellites is a possible reason for long primary cilia and retarded ciliary resorption.

The data presented in this study casts doubt on the idea that the TRAPPIII complex functions as a single entity at all time, and implicates sequential actions of individual subunits within the complex. It has also been reported that TRAPPC12 alone has a regulatory role in chromosome congression, kinetochore stability and CENP-E recruitment during mitosis (Milev et al., 2015), apart from its role in early secretory pathway as a member of TRAPPIII complex. Is it possible that TRAPPC8 and TRAPPC12 each function in ciliogenesis as individual protein, rather than members within TRAPPIII complex? These possibilities are difficult to discern at this stage, but we think a complex is more likely with the following rationale: Mammalian TRAPPII complex was reported to colocalize with centrosomal Rabin8, which is a guanine nucleotide exchange factor for the activation of Rab8 GTPase, and function in the ciliary targeting of Rabin8 during ciliogenesis. Depleting TRAPPC3 (core subunit), TRAPPC9 and TRAPPC10 (both are TRAPPII-specific) reduced centrosomal Rabin8, it was suggested that TRAPPII was responsible for Rabin8-mediated events critical for ciliogenesis (Westlake et al., 2011). However, depleting TRAPPC1, TRAPPC4, and TRAPPC5 did not reduce centrosomal Rabin8 but nonetheless negatively affected ciliogenesis, suggesting these subunits are playing a role other than the TRAPPII-Rabin8 pathway. Because TRAPPIII is the only alternative TRAPP complex found in mammalian systems, it is likely that the defect in ciliogenesis caused by depleting these subunits is due to concomitantly reduced TRAPPIII function. TRAPPC8 depletion was reported to reduce the recruitment of transfected GFP-Rabin8 to the centrosome (Schou et al., 2014), we did not find the localization of endogenous Rabin8 (by antibody staining) affected by depletion of TRAPPC8 (Figure S8), suggesting that TRAPPC8 and TRAPPC12, as subunits of TRAPPIII complex, play novel functions in ciliogenesis in way that is very different from TRAPPII. Future experiments will unravel such functions.

## Data Availability Statement

The raw data supporting the conclusions of this article will be made available by the authors, without undue reservation, to any qualified researcher.

## Author Contributions

CZ and SY designed the research. CZ performed the experiments with assistance from CL, XL, and GS. CL and SY provided reagents. CZ and SY analyzed and interpreted data. CZ wrote the manuscript, which was edited and revised by SY. All authors read and commented on the manuscript.

### Conflict of Interest

The authors declare that the research was conducted in the absence of any commercial or financial relationships that could be construed as a potential conflict of interest.

## Supplementary Material

Figure S1 TRAPPIII is localized to ER-associated structures. **(A)** Colocalization of TRAPPC8 with the indicated subcellular organelle markers by immunofluorescence. Hela cells were stained with antibodies against GM130, Sec31A, or ERGIC53. GM130 is a cis-Golgi marker. Sec31A is a subunit of COPII vesicle coat and a marker for ER exit sites (ERES), and ERGIC-53 is a marker for ER to Golgi intermediate compartments. Hela cells were treated with 10 μg/ml nocodazole for 1 h before fixation. **(B)** hTERTRPE1 cells were co-stained with antibodies against GM130, Sec31A, or ERGIC53 and with antibody against TRAPPC12. Scale bar, 10 μm. Similar results were observed in three independent experiments.

Figure S2 TRAPPIII regulates ciliogenesis. hTERT-RPE1 cells depleted TRAPPC8 or TRAPPC12 were subjected to serum starvation for 48 h. The primary cilia were stained with axoneme marker AC-tubulin (green) and cilium membrane marker ARL13B (red). Scale bar, 10 μm. Similar results were observed in three independent experiments.

Figure S3 Mass spectrometry identification of oral-facial-digital syndrome 1 protein as a binding protein to NTAP-TRAPPC12.

Figure S4 TRAPPIII depletion reduces OFD1 at centriolar satellite. **(A)** Golgi, ERGIC and ER exit sites were dispersed upon depletion of TRAPPC8 or TRAPPC12. The cells were counterstained with DAPI indicate DNA/nucleus. **(B)** Depletion of TRAPPC8 or TRAPPC12 reduced OFD1 puncta. Endogenous OFD1 and Golgi were detected with OFD1 antibody and Golgi marker GM130 in hTERT-RPE1 cells. **(C)** hTERT-RPE1 cells were incubated with 5 μg/ml of Brefeldin (BFA) for 3 h. Washed, fixed and stained for GM130 and OFD1. **(D)** Quantitative analysis of OFD1 puncta. Scale bar, 10 μm. Similar results were observed in three independent experiments.

Figure S5 Depletion of PCM1 reduces OFD1 signal at the centriolar satellites. **(A)** hTERT-RPE1 cells were depleted of siFFL or depleted of PCM1 (siPCM1) or OFD1 (siOFD1) with siRNA oligonucleotides for 72 h. The efficiency of depletion was assessed by immunoblotting for the indicated proteins including TRAPPIII components, TRAPPC8 and TRAPPC12. **(B)** Depletion of PCM1 reduced OFD1 at centriolar satellites. OFD1 was co-stained with centrosome marker γ-Tubulin. Scale bar, 10 μm. **(C)** Depletion of OFD1 leads to the dispersal of centriolar satellites. hTERT-RPE1 cells were FFL-depleted (siFFL) or depleted of OFD1 (siOFD1) with siRNA oligonucleotides for 72 h. OFD1 was co-stained with PCM1. Scale bar, 10 μm. Similar results were observed in three independent experiments.

Figure S6 PCNT and recruitment of BBSome into cilium are not affected by TRAPPIII depletion. **(A)** Depletion of PCM1 reduced pericentrin (PCNT) signals. **(B)** Quantitative analysis of PCNT. The intensities of fluorescence were measured by image J. siFFL, *n* = 40; siPCM1, *n* = 50. Mean ± SEM, ^*^*p* < 0.05, ^***^*p* < 0.01, two tailed unpaired *T*-test. Scale bar, 10 μm. **(C)** Confocal images and **(D)** quantitative analysis of PCNT in depleted of TRAPPC8 or TRAPPC12 hTERT-RPE1 cells. Scale bar, 10 μm. **(E)** Confocal images and **(F)** quantitative analysis of hTERT-RPE1 cells depleted TRAPPC8 or TRAPPC12 were subjected to serum starvation for 48 h. The primary cilia were stained with AC-tubulin (green) and BBSome component BBS9 (red). Scale bar, 10 μm. The percentage of cells with cilia that weree positive with BBS9 were quantified. The intensities of fluorescence were measured by imageJ. siFFL, *n* = 40; siTRAPPC8, *n* = 40; siTRAPPC12, *n* = 60. Mean ± SEM, ^*^*p* < 0.05, ^***^*p* < 0.01, two tailed unpaired *T*-test. Scale bar, 10 μm. Similar results were observed in three independent experiments.

Figure S7 TRAPPC12 regulates localization of BBS4 at centriolar satellites. **(A)** TRAPPC12^−/−^ but not TRAPPC8^−/−^ HEK293T cells shows dispersed BBS4 signals. **(B)** Quantitative analysis of BBS4 puncta was carried out by measuring the intensities of fluorescence in a 4 μm^2^ circular area around the centrosome by image J. Wild type, *n* = 48; TRAPPC8^−/−^, *n* = 50; TRAPPC12^−/−^, *n* = 40. Mean ± SEM, ^*^*p* < 0.05, no matching or pairing one-way ANOVA.

Figure S8 Depletion of TRAPPC8 does not reduce Rabin8 at the basal body. Confocal images of Rabin8 and γ-Tubulin staining of hTERT-RPE1 cells depleted TRAPPC8 were subjected to serum starvation for 1 h. Scale bar, 10 μm. Similar results were observed in three independent experiments. Efficiency of TRAPPC8 depletion is shown in the lower panels.
