# Supplementary figures and images for: Distinct Roles of TRAPPC8 and TRAPPC12 in Ciliogenesis via Their Interactions With OFD1
Source: Front Cell Dev Biol. 2020 Mar 17;8:148. doi: 10.3389/fcell.2020.00148 (PMC7090148; doi:10.3389/fcell.2020.00148)

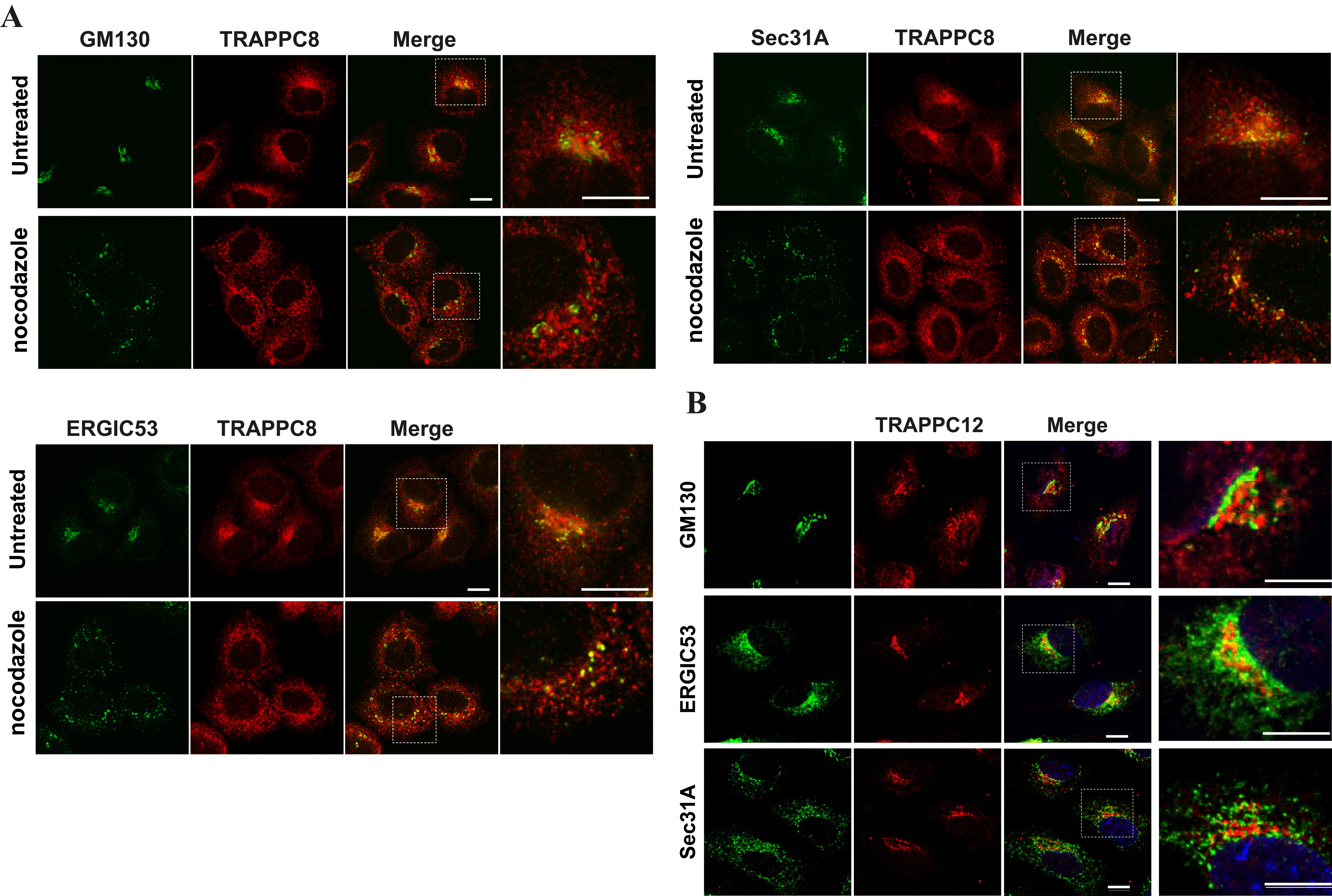

Supplement: Figure S1 — TRAPPIII is localized to ER-associated structures. (A) Colocalization of TRAPPC8 with the indicated subcellular organelle markers by immunofluorescence. Hela cells were stained with antibodies against GM130, Sec31A, or ERGIC53. GM130 is a cis-Golgi marker. Sec31A is a subunit of COPII vesicle coat and a marker for ER exit sites (ERES), and ERGIC-53 is a marker for ER to Golgi intermediate compartments. Hela cells were treated with 10 μg/ml nocodazole for 1 h before fixation. (B) hTERTRPE1 cells were co-stained with antibodies against GM130, Sec31A, or ERGIC53 and with antibody against TRAPPC12. Scale bar, 10 μm. Similar results were observed in three independent experiments. [file Image_1.JPEG]

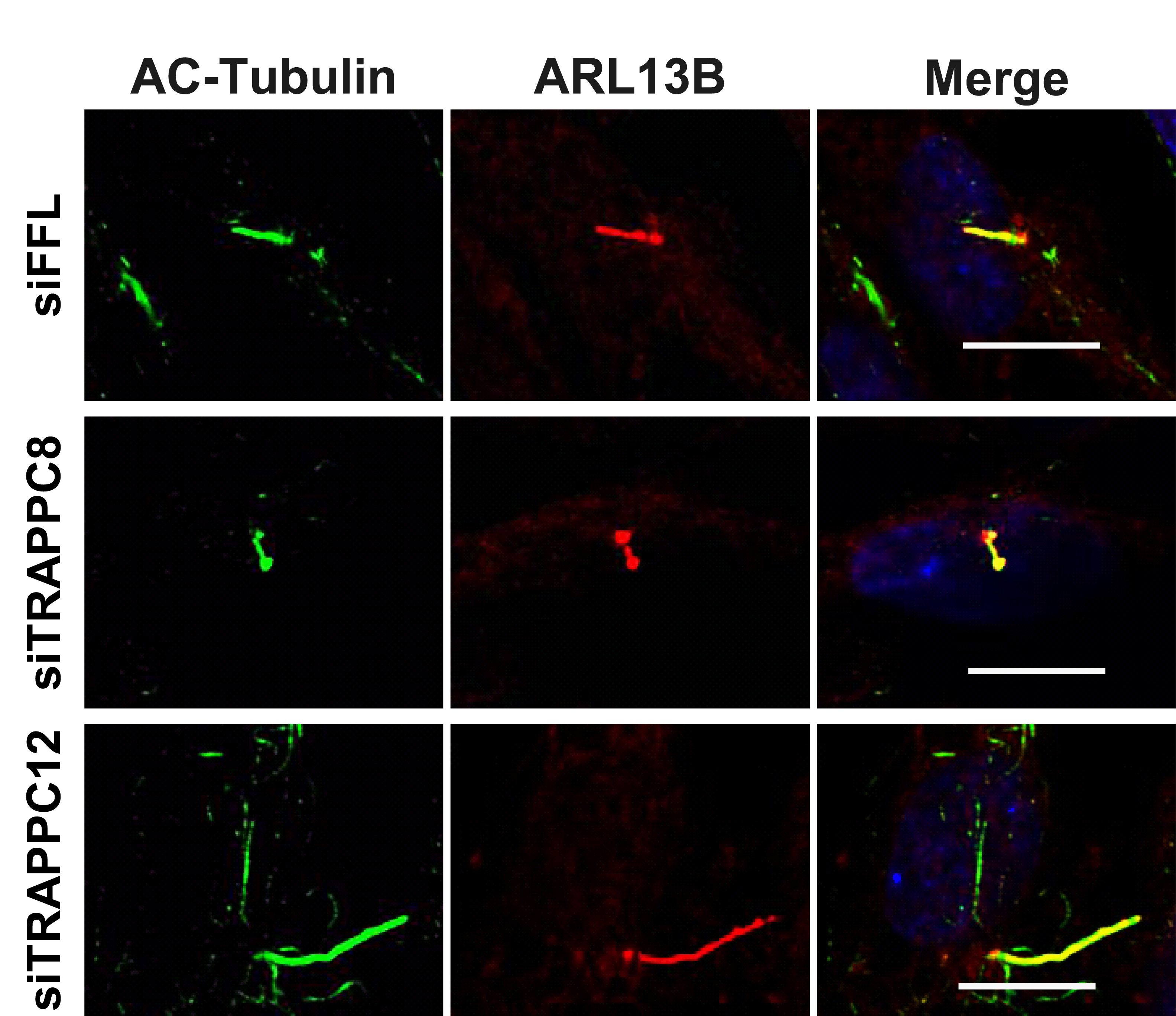

Supplement: Figure S2 — TRAPPIII regulates ciliogenesis. hTERT-RPE1 cells depleted TRAPPC8 or TRAPPC12 were subjected to serum starvation for 48 h. The primary cilia were stained with axoneme marker AC-tubulin (green) and cilium membrane marker ARL13B (red). Scale bar, 10 μm. Similar results were observed in three independent experiments. [file Image_2.JPEG]

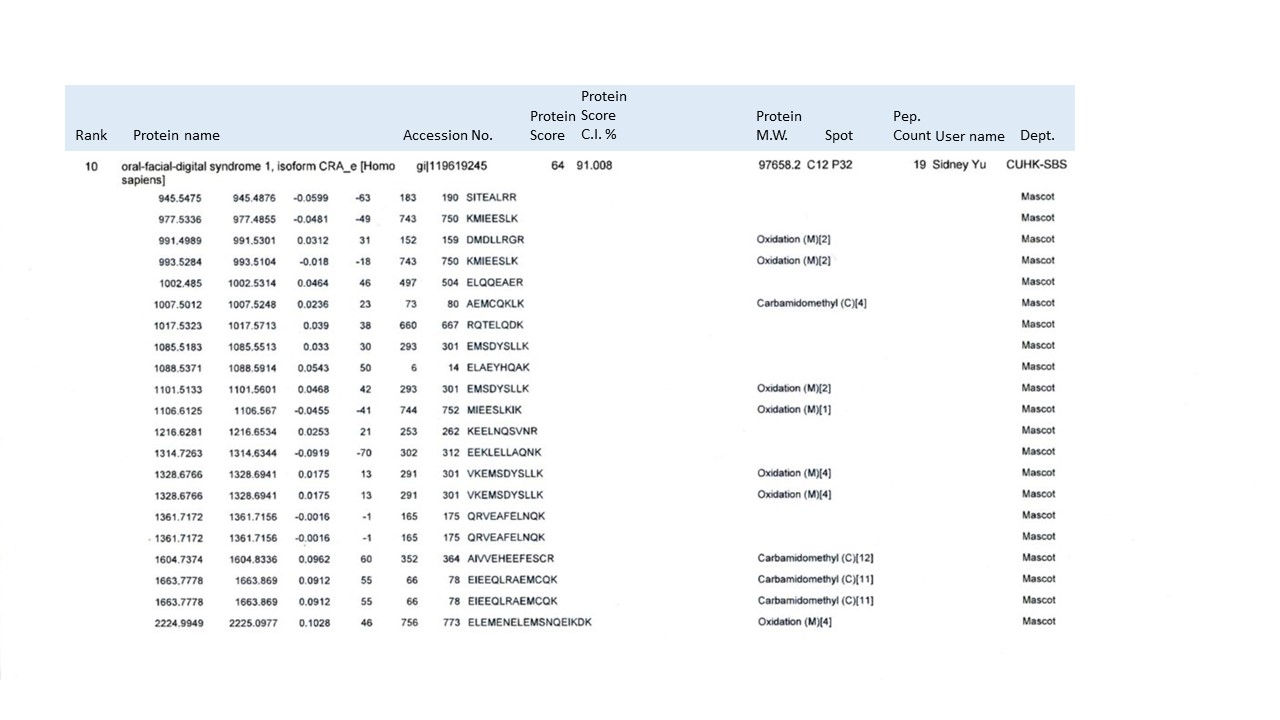

Supplement: Figure S3 — Mass spectrometry identification of oral-facial-digital syndrome 1 protein as a binding protein to NTAP-TRAPPC12. [file Image_3.JPEG]

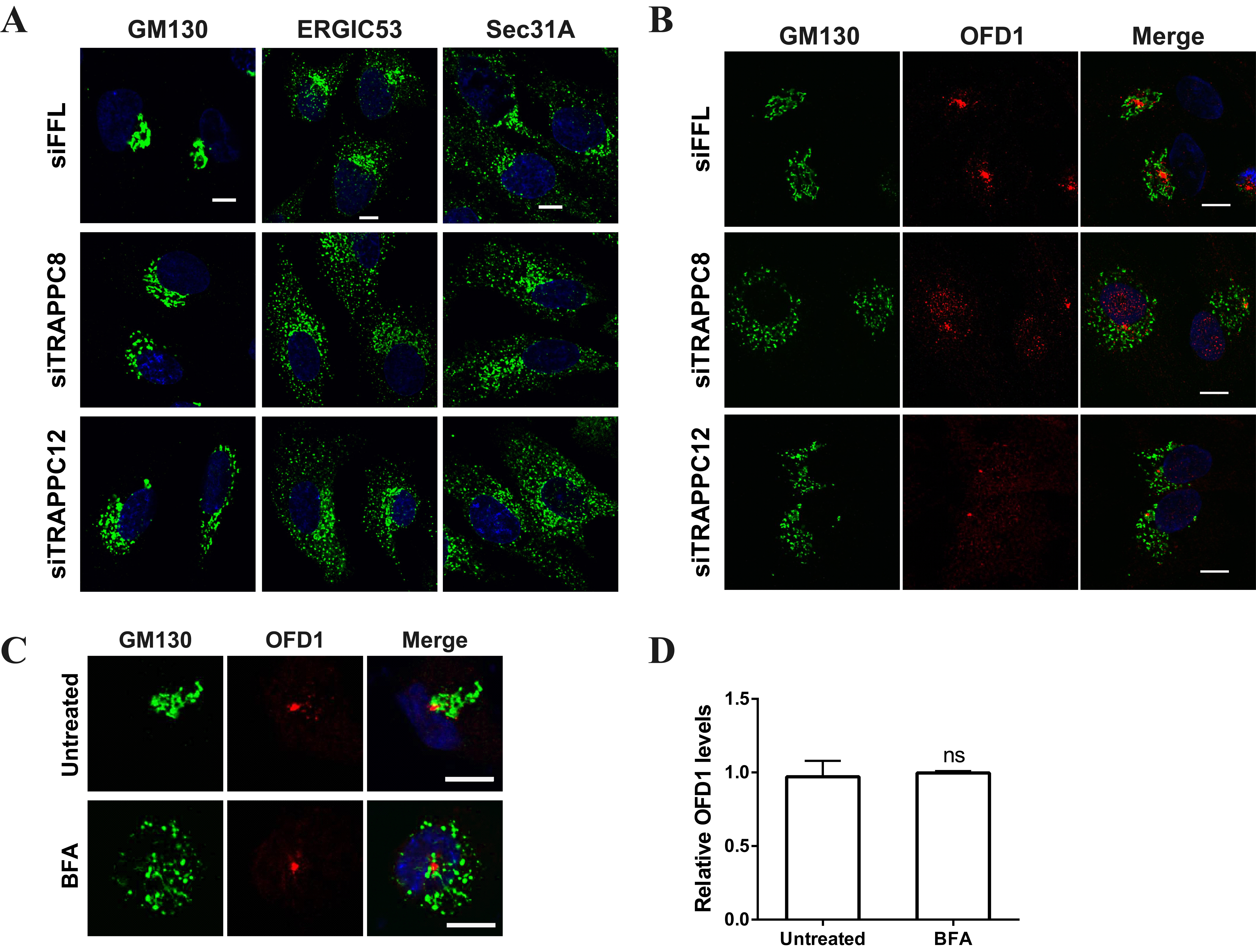

Supplement: Figure S4 — TRAPPIII depletion reduces OFD1 at centriolar satellite. (A) Golgi, ERGIC and ER exit sites were dispersed upon depletion of TRAPPC8 or TRAPPC12. The cells were counterstained with DAPI indicate DNA/nucleus. (B) Depletion of TRAPPC8 or TRAPPC12 reduced OFD1 puncta. Endogenous OFD1 and Golgi were detected with OFD1 antibody and Golgi marker GM130 in hTERT-RPE1 cells. (C) hTERT-RPE1 cells were incubated with 5 μg/ml of Brefeldin (BFA) for 3 h. Washed, fixed and stained for GM130 and OFD1. (D) Quantitative analysis of OFD1 puncta. Scale bar, 10 μm. Similar results were observed in three independent experiments. [file Image_4.JPEG]

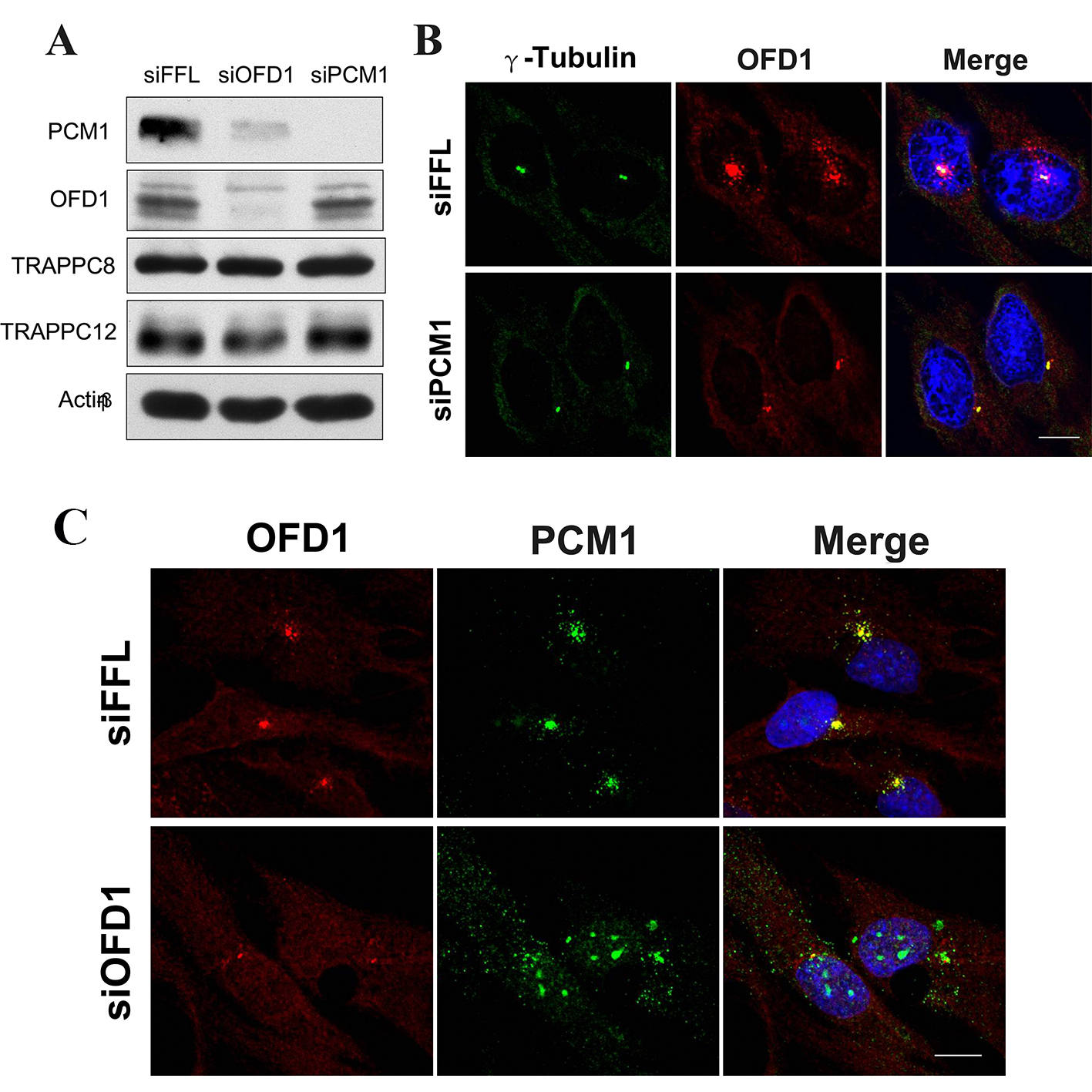

Supplement: Figure S5 — Depletion of PCM1 reduces OFD1 signal at the centriolar satellites. (A) hTERT-RPE1 cells were depleted of siFFL or depleted of PCM1 (siPCM1) or OFD1 (siOFD1) with siRNA oligonucleotides for 72 h. The efficiency of depletion was assessed by immunoblotting for the indicated proteins including TRAPPIII components, TRAPPC8 and TRAPPC12. (B) Depletion of PCM1 reduced OFD1 at centriolar satellites. OFD1 was co-stained with centrosome marker γ-Tubulin. Scale bar, 10 μm. (C) Depletion of OFD1 leads to the dispersal of centriolar satellites. hTERT-RPE1 cells were FFL-depleted (siFFL) or depleted of OFD1 (siOFD1) with siRNA oligonucleotides for 72 h. OFD1 was co-stained with PCM1. Scale bar, 10 μm. Similar results were observed in three independent experiments. [file Image_5.JPEG]

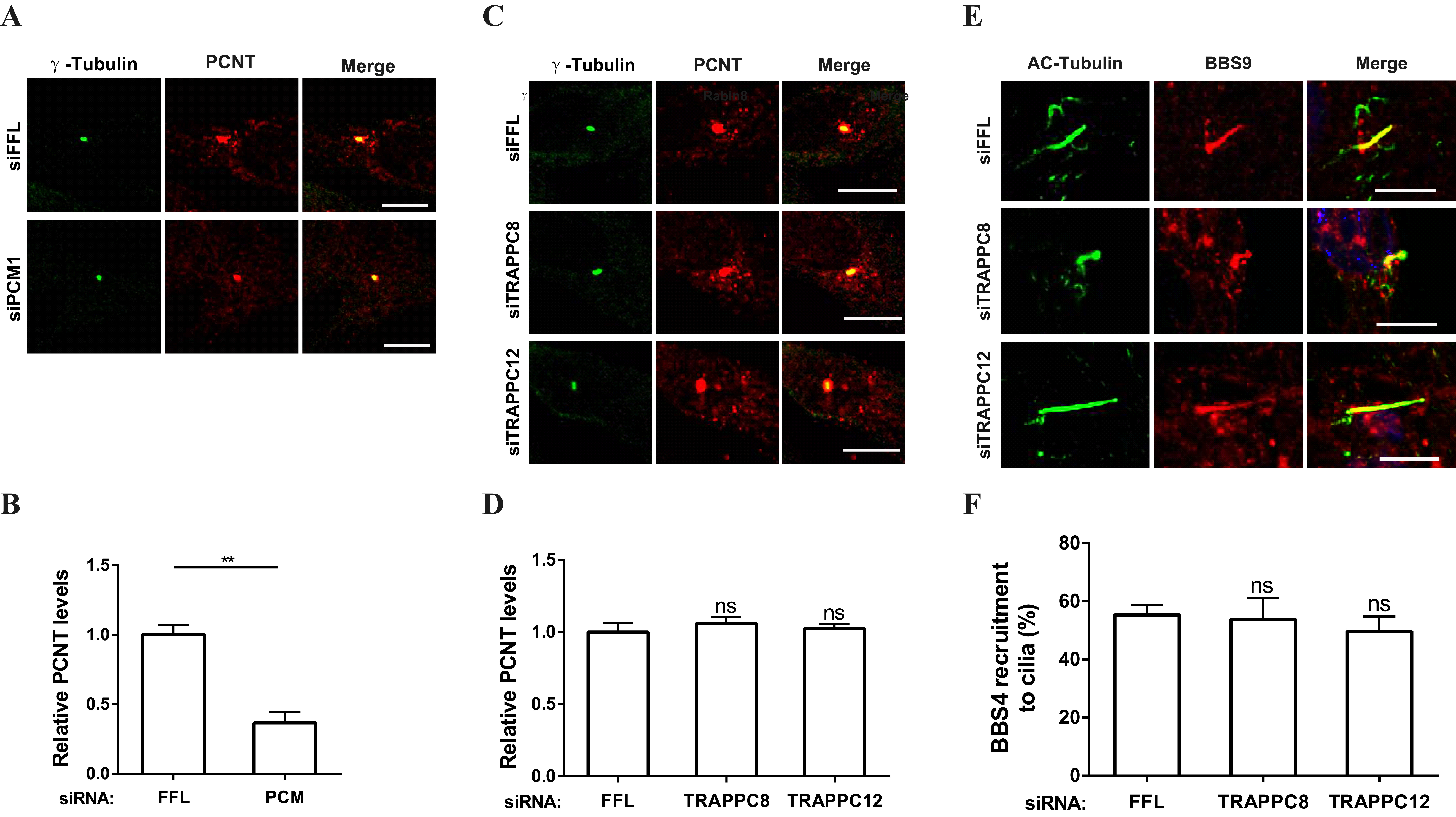

Supplement: Figure S6 — PCNT and recruitment of BBSome into cilium are not affected by TRAPPIII depletion. (A) Depletion of PCM1 reduced pericentrin (PCNT) signals. (B) Quantitative analysis of PCNT. The intensities of fluorescence were measured by image J. siFFL, n = 40; siPCM1, n = 50. Mean ± SEM, *p < 0.05, ***p < 0.01, two tailed unpaired T-test. Scale bar, 10 μm. (C) Confocal images and (D) quantitative analysis of PCNT in depleted of TRAPPC8 or TRAPPC12 hTERT-RPE1 cells. Scale bar, 10 μm. (E) Confocal images and (F) quantitative analysis of hTERT-RPE1 cells depleted TRAPPC8 or TRAPPC12 were subjected to serum starvation for 48 h. The primary cilia were stained with AC-tubulin (green) and BBSome component BBS9 (red). Scale bar, 10 μm. The percentage of cells with cilia that weree positive with BBS9 were quantified. The intensities of fluorescence were measured by imageJ. siFFL, n = 40; siTRAPPC8, n = 40; siTRAPPC12, n = 60. Mean ± SEM, *p < 0.05, ***p < 0.01, two tailed unpaired T-test. Scale bar, 10 μm. Similar results were observed in three independent experiments. [file Image_6.JPEG]

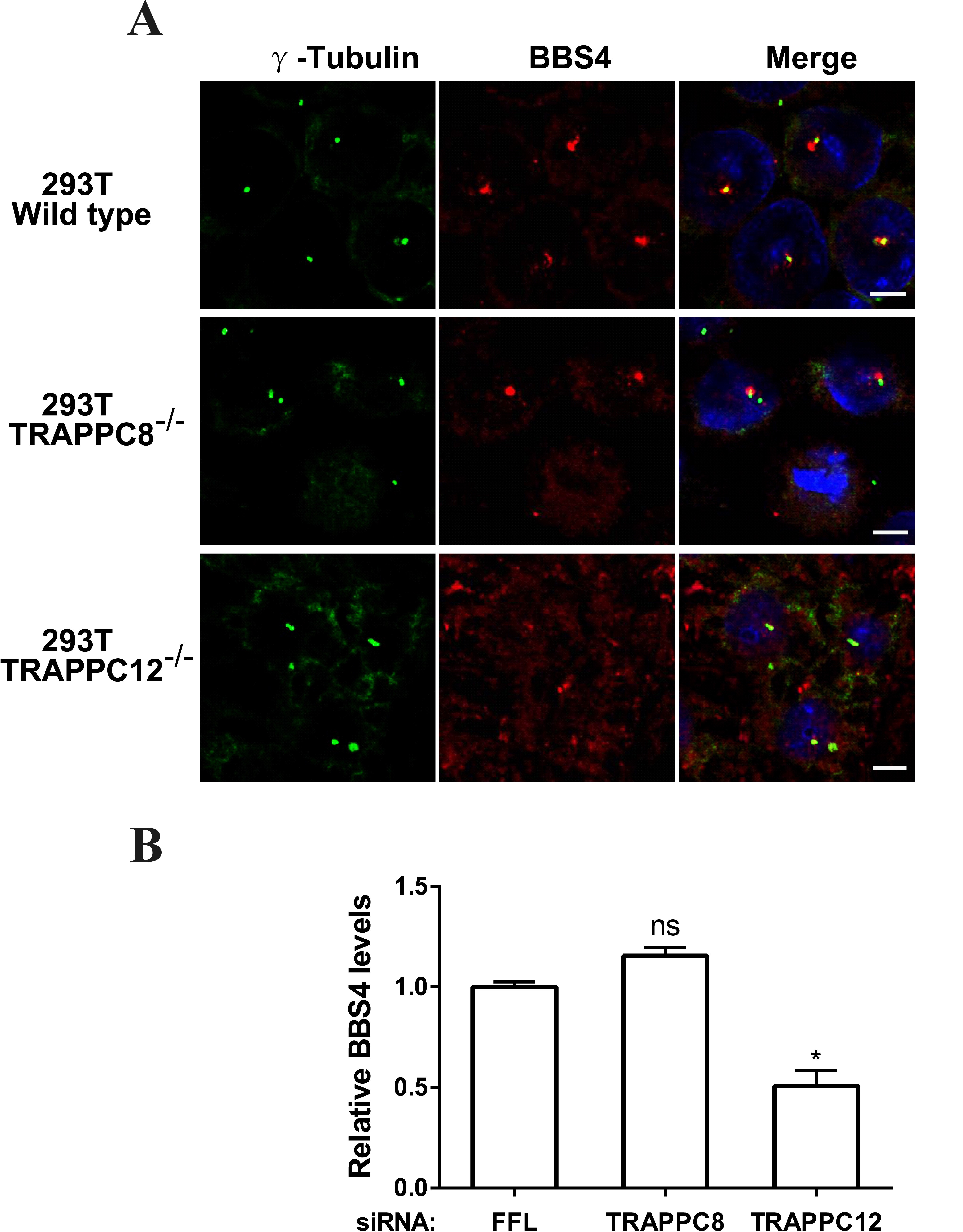

Supplement: Figure S7 — TRAPPC12 regulates localization of BBS4 at centriolar satellites. (A) TRAPPC12−/− but not TRAPPC8−/− HEK293T cells shows dispersed BBS4 signals. (B) Quantitative analysis of BBS4 puncta was carried out by measuring the intensities of fluorescence in a 4 μm2 circular area around the centrosome by image J. Wild type, n = 48; TRAPPC8−/−, n = 50; TRAPPC12−/−, n = 40. Mean ± SEM, *p < 0.05, no matching or pairing one-way ANOVA. [file Image_7.JPEG]

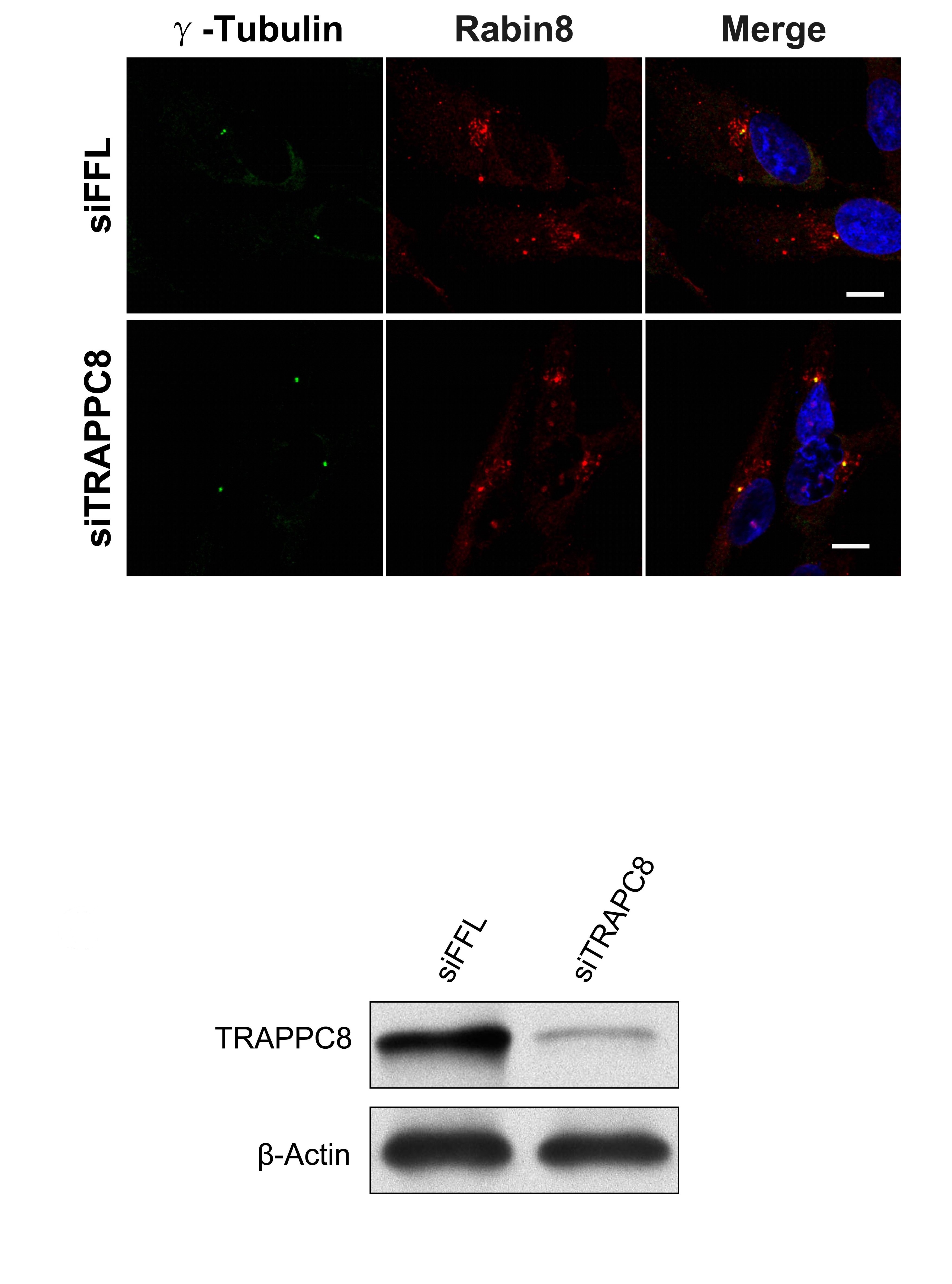

Supplement: Figure S8 — Depletion of TRAPPC8 does not reduce Rabin8 at the basal body. Confocal images of Rabin8 and γ-Tubulin staining of hTERT-RPE1 cells depleted TRAPPC8 were subjected to serum starvation for 1 h. Scale bar, 10 μm. Similar results were observed in three independent experiments. Efficiency of TRAPPC8 depletion is shown in the lower panels. [file Image_8.JPEG]
